# Inhibitory effects of proanthocyanidins from *Ribes nigrum *leaves on carrageenin acute inflammatory reactions induced in rats

**DOI:** 10.1186/1471-2210-4-25

**Published:** 2004-10-21

**Authors:** Nancy Garbacki, Monique Tits, Luc Angenot, Jacques Damas

**Affiliations:** 1 Laboratoire de Physiologie humaine, CHU, Tour 3, Université de Liège, Avenue de l'Hôpital, 3, B-4000 Sart Tilman, Belgium; 2 Laboratoire de Pharmacognosie (C.P.S.N.S.), CHU, Tour 4, Université de Liège, avenue de l'Hôpital 1, B-4000 Sart-Tilman, Belgium

## Abstract

**Background:**

The anti-inflammatory effects of proanthocyanidins (PACs), isolated from blackcurrant (*Ribes nigrum *L.) leaves, were analysed using carrageenin-induced paw oedema and carrageenin-induced pleurisy in rats.

**Results:**

Pretreatment of the animals with PACs (10, 30, 60 and 100 mg/kg, i.p.) reduced paw oedema induced by carrageenin in a dose and time-dependent manner. PACs also inhibited dose-dependently carrageenin-induced pleurisy in rats. They reduced (A) lung injury, (B) pleural exudate formation, (C) polymorphonuclear cell infiltration, (D) pleural exudate levels of TNF-α, IL-1β and CINC-1 but did not affect IL-6 and IL-10 levels. They reduced (E) pleural exudate levels of nitrite/nitrate (NOx). In indomethacin treated rats, the volume of pleural exudate was low, its content in leukocytes and its contents in TNF-α, IL-1β, IL-6 and IL-10 but not in NOx were reduced. These data suggest that the anti-inflammatory properties of PACs are achieved through a different pattern from those of indomethacin.

**Conclusion:**

These results suggest that the main mechanism of the anti-inflammatory effect of PACs mainly lies in an interference with the migration of the leukocytes. Moreover, PACs inhibited *in vivo *nitric oxide release.

## Background

Proanthocyanidins are compounds, naturally occurring in various plants, with anti-inflammatory [1,2] and anti-arthritic activities [3]. They are reported to prevent skin aging and heart diseases, they scavenge oxygen free radicals and inhibit UV radiation-induced peroxidation [4-10].

**Figure 1:**
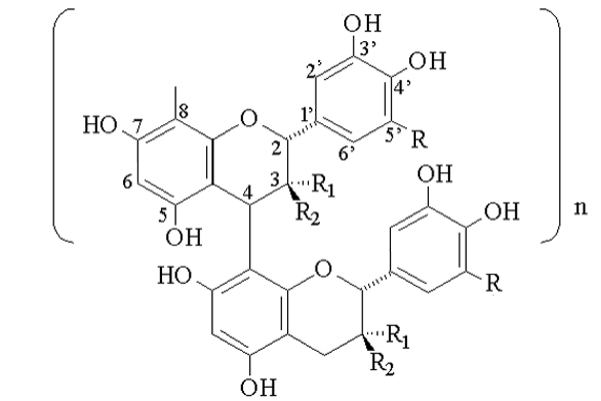
**Chemical structure of proanthocyanidins. **Where R = H, it is a procyanidin: catechin (R_1 _= H and R_2 _= OH) and epicatechin (R_1 _= OH and R_2 _= H); Where R = OH, it is a prodelphinidin: gallocatechin (R_1 _= H and R_2 _= OH) and epigallocatechin (R_1 _= OH and R_2 _= H).

We have isolated prodelphinidins and procyanidins, proanthocyanidins (PACs) from blackcurrant (*Ribes nigrum *L., Grossulariaceae) leaves which are used in European traditional medicine for the treatment of inflammatory disorders such as rheumatic diseases [11]. Majority of these compounds are water soluble monomers and oligomers (2 to 3 units) consisting of flavan 3-ol monomer units linked together by mostly C-4 to C-8 (Figure 1) and to a lesser extent C-4 to C-6 bindings. Few tetramers are also found.

Previously, we have observed that, in vitro, these compounds profoundly affect the metabolism of chondrocytes : they increase the secretion from these cells of type II collagen and proteoglycans while they decrease the secretion of prostaglandin E2 (PGE2) [12]. On the other hand, while these compounds inhibited purified cyclo-oxygenase-1 and cyclo-oxygenase-2, they did not reduce the release of thromboxane B2 and PGE2 from human in vitro stimulated platelets and neutrophils respectively [12]. Moreover, PACs might influence the contractile status of smooth muscles of blood vessels : intravenous and intraperitoneal injection of PACs induced a drop of the blood pressure without a significant bradycardia [13]. This effect counteracts the hypertensive activity of norepinephrine.

The present studies were designed to evaluate the potential anti-inflammatory activities of these compounds, *in vivo*, on carrageenin-induced paw oedema and pleurisy in rats. This latter inflammatory reaction allowed us to examine the influence of PACs not only on the exudate volume and polymorphonuclear cell accumulation but also on the release of several cytokines, IL-1β, TNF-α, IL-6, IL-10, CINC-1 and of nitric oxide (NO). These cytokines and NO are among the more important mediators involved in inflammatory processes [14-16].

## Results

### Influence of PACs on rat paw oedema

**Figure 2:**
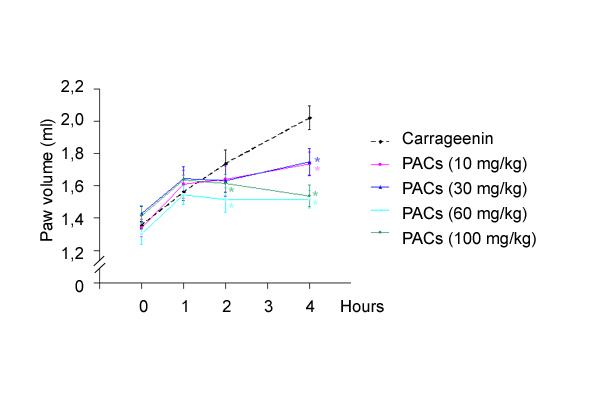
**Time course of inflammatory reaction induced by injection of carrageenin 1% in rat hind paw and its antagonism by PACs (10, 30, 60 and 100 mg/kg^-1^). **Inflammation is expressed as the increase of the rat paw volume (ml) from 0 to 4 h following injection of carrageenin. The volume of the paw was reduced by PACs at the four doses tested and the inhibition is time and dose-dependant. Each value is the mean ± s.e. mean of n = 6 experiments. *P < 0.05 *versus *carrageenin.

Carrageenin-induced oedema was significantly inhibited by PACs dose-dependently (Figure 2). This inhibitory effect was efficient from 2 h after the carrageenin injection for the two upper doses of PACs and was significative 4 h after the carrageenin administration for all doses of PACs. The maximum inhibitory effect of PACs reached 63% at 4 h after carrageenin, time of the maximum development of the oedema.

### Influence of PACs on the carrageenin-induced pleurisy

**Figure 3:**
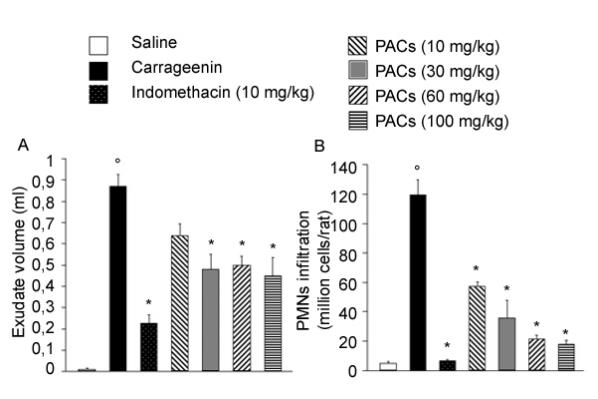
**Effect of indomethacin and PACs on carrageenin-induced pleurisy. **At 4 h after carrageenin injection, the volume of the exudate (A) was reduced by PACs (10, 30, 60 and 100 mg/kg) and indomethacin (10 mg/kg) administration. The accumulation of polymorphonuclear cells (PMNs, B) in the pleural cavity was inhibited by all tested drugs. Each value is the mean ± s.e. mean of n = 6 experiments. °P < 0.05 *versus *sham. *P < 0.05 *versus *carrageenin.

In control rats, the volume of the exudate collected 4 h after carrageenin injection reached 0.87 ± 0.18 ml per rat (n = 12). This exudate contained a large number of cells, mostly (> 95%) polymorphonuclear leukocytes (PMNs). The total leukocytes number in the exudate was 119.71 ± 29.29 × 10^6 ^per rat (Figure 3A). PACs significantly reduced the volume of the exudate in a dose-dependent relationship, showing a maximum inhibitory effect (48%) from the dose of 30 mg/kg which was not increased by the upper doses of PACs. As expected, the volume of the exudate was reduced in indomethacin-treated rats. On the other hand, PMNs infiltration (Figure 3B) was significantly inhibited by PACs in a dose-dependent way and by indomethacin.

### Effects of PACs on the release of cytokines

**Figure 4:**
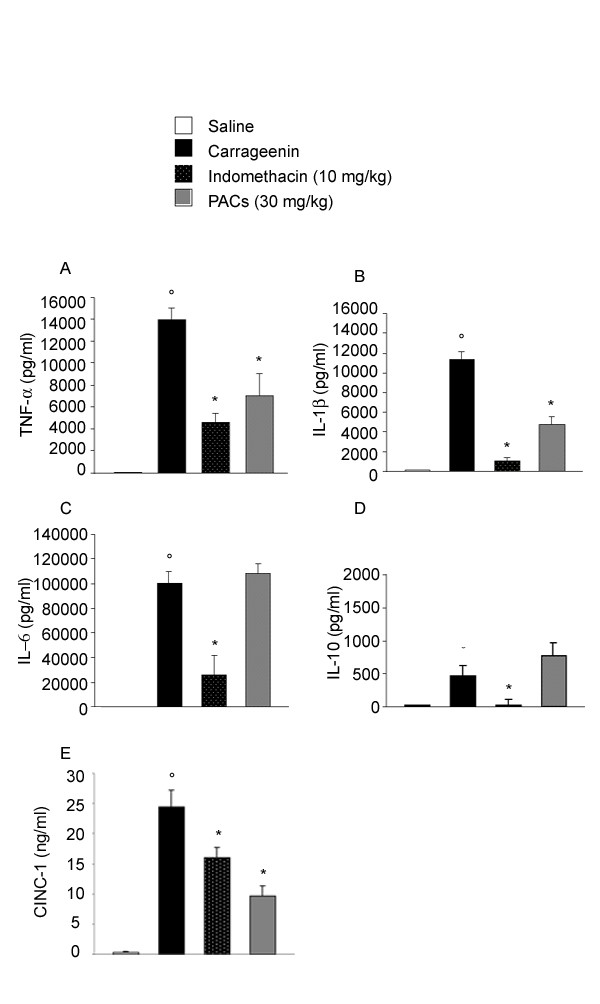
**Effect of indomethacin and PACs on cytokines release in pleural exudate. **Pleural injection of carrageenin caused by 4 h an increase in the release of the cytokines, tumor necrosis factor alpha (TNF-α, A), interleukin-1β (IL-1β, B), interleukin-6 (IL-6, C), interleukin-10 (IL-10, D) and cytokine-induced neutrophil chemoattractant-1 (CINC-1, E). TNF-α, IL-1β and CINC-1 levels were reduced by PACs, but IL-6 and IL-10 levels were not modified. Indomethacin lowered the level of all these cytokines. Each value is the mean ± s.e. mean of n = 6 experiments. °P < 0.05 *versus *sham. *P < 0.05 *versus *carrageenin.

High levels of TNF-α, IL-1β, IL-6, IL-10 and CINC-1 were found in pleural exudates induced by carrageenin (Figure 4). Indomethacin reduced the level of the five cytokines studied while PACs lowered significatively the levels of TNF-α (Figure 4A), inhibited the release of IL-1β (Figure 4B) but did not affect IL-6 levels (Figure 4C) and IL-10 production (Figure 4D). PACs also lowered significantly CINC-1 levels (Figure 4E).

### Effect of PACs on nitrite/nitrate (NOx) levels in pleural exudate

**Figure 5:**
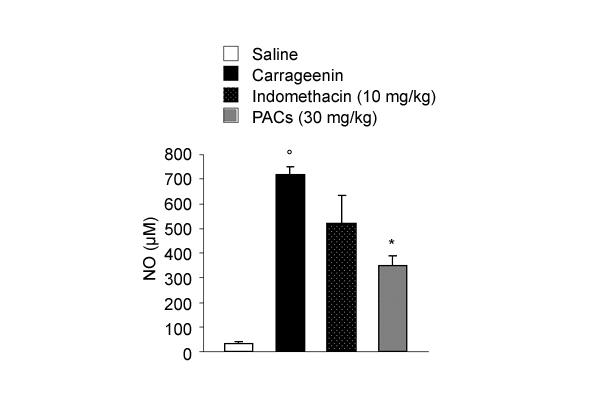
**Effect of PACs and indomethacin on NOx formation in pleural exudate. **Production of NOx release was not significantly affected by pretreatment of rats with indomethacin (10 mg/kg, intraperitoneally) while PACs caused an inhibition in NOx production. Each value is the mean ± s.e. mean of n = 6 experiments. °P < 0.05 *versus *sham. *P < 0.05 *versus *carrageenin.

The pleural exudate of carrageenin-treated rats contained a large amount of NOx (716 ± 32 μM; n = 6) (Figure 5). The amount of NOx in pleural exudate of rats treated with 10 mg/kg indomethacin was similar to the content found in the control group. On the other hand, PACs, at 30 mg/kg, significantly decreased the amounts of NOx in pleural exudate from 51%.

### Histological examination of lung sections

**Figure 6:**
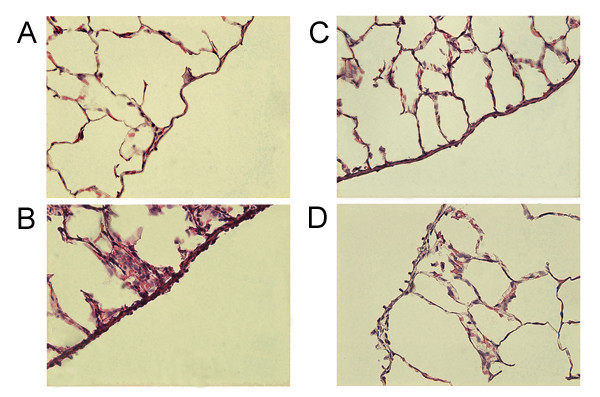
**Effect of PACs on lung injury. **When compared to lung sections taken from control animals (A), lung sections from carrageenin-treated rats (B) demonstrated interstitial haemorrhage and polymorphonuclear leukocyte accumulation. Lung sections from a carrageenin-treated rat that had received PACs (30 mg/kg) (C) or indomethacin (10 mg/kg) (D) exhibited reduced interstitial haemorraghe and a lesser cellular infiltration. Original magnification: × 125.

Histological examination of lung sections revealed significant tissue injury (Figure 6) when compared with lung sections taken from saline-treated rats (Figure 6A). Lung withdrawn from rats treated with carrageenin showed oedema, tissue injury and an extensive infiltration of the tissue by PMNs (Figure 6B). Pretreatment of rats with indomethacin (10 mg/kg, i.p.) or PACs (30 mg/kg, i.p.) showed a reduced lung injury as well as a decrease in the infiltration of PMNs (Figures 6C,6D).

## Discussion

Proanthocyanidins (PACs) from *Ribes nigrum *leaves reduced the inflammatory reactions induced by carrageenin in rats : the extent of the paw oedema was halved, the volume of the pleural exudates and its content in TNF-α, IL-1β, CINC-1 and NOx were reduced, the infiltration of leukocytes into the lungs and the accumulation of leukocytes into the pleural cavity were largely diminished.

PACs have been reported to be able to scavenge free radicals and NO [17]. This property could be an explanation of the reduction of NOx level in the pleural fluid after PACs treatment. According to Ialenti *et al *[18], during the development of carrageenin-induced pleurisy, the main role of NO is the inhibition of leukocytes migration to the inflammatory site. However, in rats pretreated with PACs, the level of NOx and of leukocytes are simultaneously reduced. This result suggests that PACs could more or less directly affect the transmigration of leukocytes.

The development of carrageenin-induced inflammatory reactions in rats results from the activation of the kinin system, the accumulation of leukocytes and the release of several mediators such as prostanoids and cytokines [19,20]. Indeed, these inflammatory reactions are greatly reduced in kininogen-deficient rats, in animals pretreated with kinin-antagonists and in leucopenic rats [19,21]. Previous studies [22] have demonstrated that PACs can reduce other inflammatory reactions such as the oedemas induced in rats by nystatin and concanavalin-A in which the kinin system is not involved [19] but in which leukocytes play a major role [23]. The comparison of the major determinants of these three kinds of reactions, all inhibited by PACs, is another argument suggesting that the main target explaining the anti-inflammatory activity of PACs would be the involvement of leukocytes.

Pro-inflammatory cytokines TNF-α, IL-1β and IL-6 are sequentially released in the pleural exudates induced by carrageenin in rat [14]. These cytokines cause chemotaxis to attract granulocytes and monocytes and then, migrating leukocytes produce, in turn, further cytokines, such as TNF-α and IL-1β, and other pro-inflammatory mediators [15]. IL-6 has been proposed as a crucial mediator for the development of carrageenin-induced pleurisy and for the accumulation of leukocytes in the inflammatory site. Indeed, in carrageenin-induced pleurisy in IL-6 knock-out mice, the degree of plasma exudation, leukocyte migration and the release of TNF-α and IL-1β were greatly reduced. Moreover, a positive feedback plays an important part in the development of the oedema as levels of TNF-α and IL-1β are attenuated in IL-6 knock-out mice [24]. PACs did not affect the level of IL-6 and of IL-10, an anti-inflammatory cytokine, but reduced the pleural content of TNF-α, IL-1β and leukocytes. This result indicates that the release of IL-6 does not depend on the presence of leukocytes, of TNF-α and IL-1β on one hand, and, on the other hand, suggest that the main target of PACs would be the accumulation of leukocytes and the associated release of inflammatory mediators.

TNF-α plays an important role in promoting and amplifying lung inflammation through the release of chemotactic factors such as CINC-1 (rat IL-8), an important mediator that promotes the migration of neutrophils [25] and oesinophils [26]. CINC-1 can increase the expression of LFA-1 integrin on rat neutrophils [27] and because expression of leukocyte adhesion molecules such as E-selectin is dependent on CINC [28], the inhibition of CINC-1 levels in pleural exudates by PACs may exert both direct and indirect effects on neutrophil vascular adhesion and extravascular migration. PACs probably acts by disrupting TNF-α, IL-1β, CINC-1 and PMNs accumulation pathways. One of the mechanism for the anti-inflammatory effect of PACs may be attenuation of the migration of PMNs in the exudate, because CINC-1, a representative cytokine for PMNs migration in rats, is suppressed by PACs in parallel with PMNs number dose-related fashion. Although, clarification for the precise mechanism would remain in future study.

Recently, grape seed proanthocyanidins have been demonstrated to reduce the expression of soluble adhesion molecules, ICAM-1, VCAM-1 and E-selectin in the plasma of systemic sclerosis patients [29]. The same compounds have been shown to inhibit TNF-α-induced V-CAM-1 expression in human umbilical vein endothelial cells cultures [30]. A possible mechanism of the anti-inflammatory effect of PACs would be an interference with the expression or the effect of adhesion molecules. This interference would result in a reduction of polymorphonuclear cell migration and subsequently in a reduction of the release of pro-inflammatory factors such as TNF-α and IL-1β.

Injection of carrageenin into the pleural cavity induces the accumulation of leukocytes, a release of cytokines, the expression of inducible NO synthase and of cyclo-oxygenase-2, and thus the release of large amounts of nitric oxide and of prostanoids [16]. The inhibitory effect of PACs on the accumulation of leukocytes and on the release of TNF-α and IL-1β could have resulted in a decrease in the induction of inducible NO-synthase and of cyclo-oxygenase-2 and finally of plasma exudation.

Comparatively, some animals have been treated with indomethacin. The inhibitory effect of this well-known non-steroidal anti-inflammatory drug is larger than that obtained with PACs. Indomethacin greatly reduced plasma exudation, nearly suppressed the accumulation of leukocytes and decreased the levels of the cytokines while, it did not modify the pleural content of NOx. Indomethacin is known to inhibit the cyclooxygenase-1 and -2 responsible of the release of PGE_2 _production. The peak of cyclooxygenase-2 activity measured by prostanoid levels in carrageenin-induced pleural exudates spreads from 2 to 6 h after irritant injection [31,32]. Both IL-6 and IL-10 release are, in part, stimulated by PGE_2 _[33,34]. An inhibition of PGE_2 _production by high doses of indomethacin could result in a downregulation of IL-6 and IL-10 production [35,36]. Moreover, Cuzzocrea *et al *[24], using carrageenin-induced pleurisy in IL-6 knock out mice, showed that IL-1β and TNF-α production in the pleural exudates is, at least, partly IL-6 dependent. Our results showing a reduction in the levels of IL-1β, TNF-α, IL-6, IL-10 and CINC-1 by indomethacin four hours after the induction of the pleurisy, could be mainly explained through the inhibition of PGE_2 _and IL-6 pathways.

## Conclusions

In conclusion, we have shown that proanthocyanidins isolated from *Ribes nigrum *leaves interfere with the accumulation of circulating leukocytes, associated with a reduction of pro-inflammatory factors such as TNF-α, IL-1β and CINC-1, a decrease of NOx level and a decrease in plasma exudation.

## Methods

### Animals

We used male Wistar rats, weighing 250 – 300 gm. The animals were maintained on a standard laboratory diet with free access to water. The experiments were conducted as approved by the Animal Ethics Committee of the University of Liège, Belgium.

### Paw oedema

Rats were pretreated with an intraperitoneal administration of saline or PACs (10, 30, 60 and 100 mg/kg). Thirty minutes later, lambda carrageenin, (0.1 ml, 10 mg/ml) was injected into the plantar region of the right hind paw. Each experimental group contained six animals. Paw volume was measured using a water plethysmometer (Ugo Basile) before and 1 h, 2 h and 4 h after the injection of carrageenin. After 4 h, the animals were anaesthetized with a large dose of sodium pentobarbital (80 mg/kg).

### Carrageenin-induced pleurisy

Rats were pretreated with an intraperitoneal injection of saline, PACs (10, 30, 60 or 100 mg/kg) or indomethacin (10 mg/kg) 30 min before the intrapleural injection of the irritant. They were then anaesthetized with ketamine HCl (75 mg/kg) and carrageenin (0.2 ml, 10 mg/ml) or saline (0.2 ml) was administered into the right pleural cavity. Each experimental group contained 6 animals. Four hours later, the animals were anaesthetized with sodium pentobarbital (80 mg/kg). The chest was carefully opened and the pleural cavity rinsed with 2.0 ml saline solution containing heparin (5 U/ml). Exudates and washing solutions were removed by aspiration and the total volume measured. Exudates with blood were rejected. Exudates were aliquoted and kept frozen at -32°C.

After removal of the exudates, lungs were withdrawn and fixed for one week under 30 cm pressure with 10% formaldehyde aqueous solution containing 0.480 M Na_2_HPO_4 _and 0.187 M KH_2_PO_4 _(pH 7.2) at room temperature. They were then dehydrated by graded ethanol and embedded in Paraplast. Tissue sections (thickness 7 μm) were deparaffinized with UltraClear, stained with hematoxylin-eosine and examined using light microscopy.

The volume of the exudates was calculated by subtracting the volume of the washing solution (2.0 ml) from the total volume recovered. A sample of each exudate was diluted in phosphate buffer and total leukocyte count was performed using a hemocytometer.

The levels of IL-1β, TNF-α, IL-6 and IL-10 in the exudates were measured using a colorimetric commercial ELISA kit (Biosource, Nivelles, Belgium) with a lower detection limit of 4, 3, 8 and 5 pg/ml, respectively. The levels of CINC-1 in the exudates were measured using a colorimetric commercial ELISA kit (Amersham Biosciences, Freiburg, Germany) with a lower detection limit of 0.49 pg/ml.

The amount of NOx (nitrite/nitrate) present in the exudates was determined using a microplate assay method (Calbiochem, Leuven, Belgium) based on Griess reaction after reduction of NO_3 _^- ^to NO_2 _^- ^with a lower detection limit of 1 μM.

### Extraction and purification of proanthocyanidins

Proanthocyanidins from *Ribes nigrum *leaves were extracted and isolated according to a previously described method [37]. A voucher sample (RN 210590) has been deposited in the Pharmaceutical Institute of Liège, Belgium. Briefly, leaves were powdered separately and then extracted at room temperature with acetone (70 % v/v in water). The acetone was removed under vacuum at 40°C. The resulting aqueous solution was freeze-dried. Isolation was carried out by MPLC on reversed-phase RP8 with water-acetone (9:1) to obtain a total proanthocyanidin-enriched fraction (PACs).

### Materials

We used ketamine-HCl from Pfizer (Bruxelles, Belgium), sodium pentobarbital from Ceva (Bruxelles, Belgium) and heparin from B. Braun Medicals (Diegem, Belgium). PACs and lambda carrageenin (Sigma, Bornem, Belgium) were dissolved in saline. Indomethacin (Merck, Sharp and Dohme, Leuven, Belgium) was dissolved in Tris-HCl (0.15 M, pH 7.4).

### Statistical evaluation

Results are given as mean ± standard error of the mean (s.e. mean) of N observations. For the oedema paw studies, a Mixed Procedure SAS (normal distribution) was used to compare difference of least square means. For the pleurisy studies, data sets were examined by one-way analysis of variance (ANOVA) followed by a Scheffe post-hoc test. A *P*-value of less than 0.05 was considered significant.

## Authors' contributions

NG carried out PACs isolation, animal experimentation, immunoassays, lung sections and statistical analysis. MT coordinated and participated to the PACs isolation. LA coordinated the PACs isolation. JD participated in animal experimentation, conceived of the study and participated in its design and coordination.
